# Large Size Color-tunable Electroluminescence from Cationic Iridium Complexes-based Light-emitting Electrochemical Cells

**DOI:** 10.1038/srep27613

**Published:** 2016-06-09

**Authors:** Qunying Zeng, Fushan Li, Tailiang Guo, Guogang Shan, Zhongmin Su

**Affiliations:** 1 Institute of Optoelectronic Technology, Fuzhou University, Fuzhou 350002, People’s Republic of China; 2 Institute of Functional Material Chemistry, Faculty of Chemistry, Northeast Normal University, Changchun, Jilin 130024, People’s Republic of China

## Abstract

Solution-processable light-emitting electrochemical cells (LECs) with simple device architecture have become an attractive candidate for application in next generation lighting and flat-panel displays. Herein, single layer LECs employing two cationic Ir(III) complexes showing highly efficient blue-green and yellow electroluminescence with peak current efficiency of 31.6 cd A^−1^ and 40.6 cd A^−1^, respectively, have been reported. By using both complexes in the device, color-tunable LECs with a single spectral peak in the wavelength range from 499 to 570 nm were obtained by varying their rations. In addition, the fabrication of efficient LECs was demonstrated based on low cost doctor-blade coating technique, which was compatible with the roll to roll fabrication process for the large size production. In this work, for the first time, 4 inch LEC devices by doctor-blade coating were fabricated, which exhibit the efficiencies of 23.4 cd A^−1^ and 25.4 cd A^−1^ for the blue-green and yellow emission, respectively. The exciting results indicated that highly efficient LECs with controllable color could be realized and find practical application in large size lighting and displays.

The light-emitting electrochemical cells (LECs) were firstly reported by Pei *et al*. in ref. 1. In comparison with the conventional multi-layer organic light-emitting devices (OLEDs), LECs have a simple architecture and do not rely on air-sensitive charge injection layers or metals for electron injection^1^,^2^,^3^,^4^. LECs employ a sandwich device structure which can be easily fabricated from solution process, consisting of a light-emitting layer with ionic species between two electrodes^5^,^6^,^7^. In addition, the presence of mobile ions facilitates the formation of ionic junctions, which lowers the barrier for electron and hole injection and causes LECs devices independent of the work function of cathode materials^1^,^2^. These characteristics make LECs simple preparation and low cost, and LECs are thus superior candidate for the applications in next generation lighting and flat-panel displays.

At present, most of the research of LECs can be divided into four categories: conjugated light-emitting polymers LECs (PLECs)^1^,^8^,^9^,^10^,^11^, ionic transition-metals complexes LECs (iTMC-LECs)^6^,^12^,^13^,^14^,^15^, quantum dots LECs (QD-LECs)^16^,^17^ and non-ionic small molecules LECs (SM-LECs)^18^. Among the iTMCs, cationic Ir(III) complexes in terms of their short excited-state lifetimes, tunable color, easy synthesis and purification, good solubility and high photoluminescence quantum yields, have drawn much attention and emerged as promising candidates for LECs applications. In 2004, the first LEC based on ionic Ir(III) complexes was reported by Slinker *et al*.^6^. To realize high-efficiency full-color LEC devices, the optimization of the cationic Ir(III) complexes structure, LEC device architecture and preparation process is highly desired. However, the luminescent quenching always happens because of the interaction between closely packed molecules in the film, which limits the development of high-efficiency and long lifetime devices. On the other hand, the method of fabricating LECs usually uses spin-coating approach, however, it is not compatible with large size production processes and flexible substrate. Alternatively, doctor-blade coating is a simple, cost-efficient, and roll to roll compatible process for optoelectronic device fabrication^19^,^20^,^21^. Compared with the spin coating, the advantages of doctor-blade coating are that it has a high utilization rate of materials and can fabricate large scale and continuous processing of thin film. It is thus expected that the large-scale LECs as an alternative emissive device can be fabricated with all-solution processing by doctor-blade coating. To date, the fabrications of LECs with doctor-blade coating are still rarely reported. Achieving all-solution processed and large-size LECs still remains a great challenge.

In this paper, we report the highly efficient blue-green and yellow LECs based on cationic Ir(III) complexes [Ir(dfppz)_2_(Metz)]PF_6_ (complex **B**) and [Ir(ppy)_2_(Metz)]PF_6_ (complex **Y**) where dfppz, ppy and Metz are 1-(2,4-difluorophenyl)-1*H*-pyrazole, 2-phenyl pyridine and 2-(5-methyl-2-phenyl-2*H*-1,2,4-triazol-3-yl)pyridine, respectively. Devices were fabricated with single light-emitting layer structure using the following configuration: indium tin oxide (ITO)/poly(3,4-ethylenedioxythiophene)-poly (styrenesulfonate) (PEDOT:PSS)/light-emitting layer/Al. The impact of molecular structure of the complexes on the device performance is investigated. Moreover, by varying ratios of blue-green and yellow emitting cationic Ir(III) complexes, the color-tunable LECs devices with a single spectral peak in electroluminescence (EL) spectra were achieved. The devices using hybrid complexes are found to exhibit high current efficiencies in a wide wavelength range. Furthermore, we report a technique to fabricate high efficiency large area LECs using a doctor-blade coating.

## Experimental details

### Synthesis of [Ir(dfppz)_2_ Metz]PF_6_ and [Ir(ppy)_2_ Metz]PF_6_

Complexes **B** and **Y** were synthesized with the treatment of the dichloro-bridged diiridium complex [Ir(dfppz)_2_Cl]_2_ and [Ir(ppy)_2_Cl]_2_ with the ancillary ligands Metz by a bridge-splitting reaction in dichloromethane–methanol (2:1, V:V) under the dark condition^22^. After cooling to room temperature, the mixture was filtrated, and then an excess of solid KPF_6_ was added and stirred for another 0.5 h at room temperature. The solvent was removed under reduced pressure and the residue was purified by silica gel column chromatography to yield the target complexes.

The molecular weights of the target complexes were tested by using matrix-assisted laser desorption-ionization time-of-flight (MALDI-TOF) mass spectrometry, respectively. UV-vis absorption spectra were recorded on a Hitachi U3030 spectrometer. The excited-state lifetime were measured on a transient spectrofluorimeter (Edinburgh FLS920) with time-correlated single-photon counting technique. The photoluminescence quantum yields (PLQYs) of the neat film were measured in an integrating sphere.

### Fabrication of LECs

ITO-coated glass substrates with a sheet resistance of (20 ± 5 Ω/sq) were cleaned by ultrasonic treatment for 20 min sequentially in acetone, ethanol, deionized water and then dried in an oven. Then, the ITO substrates were treated with UV-ozone for 10 min to improve the hydrophilicity. The PEDOT:PSS solutions was spin-coated onto ITO-coated glass substrates at 3000 rpm for 40 s and annealed at 160 °C for 20 min in air, yielding a film thickness of about 50 nm. Cationic iridium complexes (20 mg) and 1-butyl-3-methylimidazolium hexafluorophosphate (BMIM-PF_6_) (10 mg) were dissolved in 1 ml CH_3_CN solutions. The solution was stirred for 3 h at room temperature before use. Light-emitting layer consisting of iridium complexes and BMIM-PF_6_ was spin coated on the top of PEDOT:PSS layer at 1500 rpm for 45 s, yielding a layer thickness of about 100–150 nm. The film was then baked at 75 °C for 30 min in air. Finally, an aluminum cathode (100 nm) was thermally evaporated in vacuum chamber below 3 × 10^−3^ Pa. The active light emitting area was 5 × 5 mm^2^.

### Fabrication of LECs by doctor-blade coating

The preparation of ITO-coated glass substrates and complexes solution is the same as that of spin coating process. In the doctor-blade coating process, the PEDOT:PSS solution was dropped onto ITO-coated glass substrate maintained at 80 °C, and swiped linearly by a steel blade at a speed of 0.5 cm/s. The film was annealed at 160 °C in air for 20 min after blade coating. The complex solution with BMIM-PF6 was dropped onto the PEDOT:PSS film, and swiped by blade at a speed of 0.7 m/s. Then, the film was annealed at 75 °C in air for 30 min. The thickness of the light-emitting layer films was controlled by complex solution concentration, scraping speed and the spacing between steel blade and the substrates. Finally, an aluminum cathode (100 nm) was thermally evaporated in vacuum chamber below 3 × 10^−3^ Pa. The active light emitting area were 5 × 5 mm^2^ and 8 × 6 cm^2^ (4 inches), respectively.

To investigate the optical and electrical properties of the as-fabricated LECs, the PL and EL spectra were recorded with a Hitachi F-4600 fluorescence spectrophotometer. The luminance intensity and CIE coordinates were measured using a Topcon SR-3A spectroradiometer. The current-voltage (I-V) and current-time (I-t) measurements were performed by using Keithley 4200 semiconductor characterization system. In addition to the deposition of Al electrodes, all fabrications and tests were carried out at room temperature in atmospheric pressure.

## Results and Discussion

**Figure 1:**
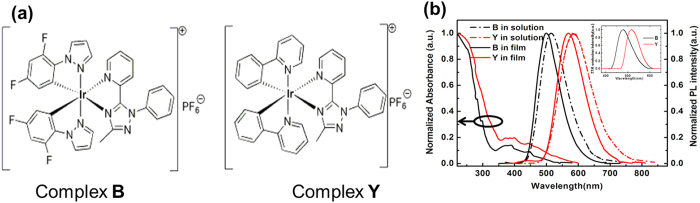
(**a**) Chemical structures of the cationic Ir(III) complexes **B** and **Y**. (**b**) Normalized absorption and PL spectra of complexes **B** and **Y** at room temperature, the inset is the emission at 77 K.

The cationic Ir(III) complexes used in this study were [Ir(dfppz)_2_Metz]PF_6_ and [Ir(ppy)_2_Metz]PF_6_, as shown in Fig. 1a. The absorption and photoluminescence (PL) spectra of complexes **B** and **Y** in CH_3_CN solutions at room temperature are shown in Fig. 1b. The detailed photophysical and electrochemical characteristics are summarized in Table 1. The intense absorption bands in the ultraviolet region of 220–350 nm are ascribed to π-π^*^ transitions from the ligands. For the cationic iridium complexes, there are three principle transitions which are observed in the excited states: metal to ligand charge transfer (^3^MLCT), ligands to ligands charge transfer (^3^LLCT), and ligand-centered (^3^LC) transitions^23^. At room temperature, both complexes show broad and featureless emission spectra in their CH_3_CN solutions and in neat films, suggesting that their emissive excited states mainly originate from ^3^MLCT and ^3^LLCT. For complexes **B** and **Y**, the maximum emission peaks appear at 516 nm, 586 nm in solutions and at 499 nm, 571 nm in films, respectively. The peak positions are different, due to the varying intensity of interaction of complexes ligand plane in different stages. The complex molecules accumulated more closely in the solid phase, which cause free rotation of molecular internal structure to be impeded^3^,^6^. Upon cooling CH_3_CN solutions to 77 K, their emission spectra undergo rigidochromic blue shifts, but still are featureless. These emission properties at 77 K further suggest that their emissions mainly take on ^3^MLCT mixed with ^3^LLCT states^24^,^25^. It is clear that the emission of complex **B** is obviously blue-shifted compared to that of complex **Y**, which is attributed the reduced highest occupied molecular orbit (HOMO) levels caused by the attachment of fluorin atoms into the cyclometalated ligands.

The cyclic voltammogram of complexes **B** and **Y** in CH_3_CN solution (Fig. S1, Supporting Information) and the redox potentials are listed in Table 1. Both complexes exhibit reversible oxidation and reduction processes in CH_3_CN solution. Such excellent redox reversibility is beneficial for their application in LECs. The reduction potential of complex **B** (−1.92 V) is close to that of complex **Y** (−1.96 V) because the reduction of the cationic Ir(III) complexes occurs on the same ancillary ligand Metz. The oxidation potential of complex **B** (1.16 V) is shifted anodically with respect to that of complex **Y** (0.79 V), indicating that the HOMO energy level of complex **B** was stabilized as compared to the complex **Y**. Therefore, the energy gap of complex **Y** should be smaller than that of complex **B**, which is in accordance with our photophysical data. The electrochemical gaps Δ*E* = *E*_*ox*_^1/2^ − *E*_*red*_^1/2^ found for complexes **B** (3.08 V) and **Y** (2.75 V) are similar to the energy gaps obtained from density functional theory (DFT) calculations (2.99 eV and 2.67 eV, respectively) (Fig. S2, Supporting Information). Our theoretical calculations also indicate that the lowest triplet (T_1_) states of studied complexes **B** and **Y** mainly originate from the excitation of HOMO to lowest unoccupied molecular orbit (LUMO), with characters of ^3^MCLT and ^3^LLCT.

**Figure 2:**
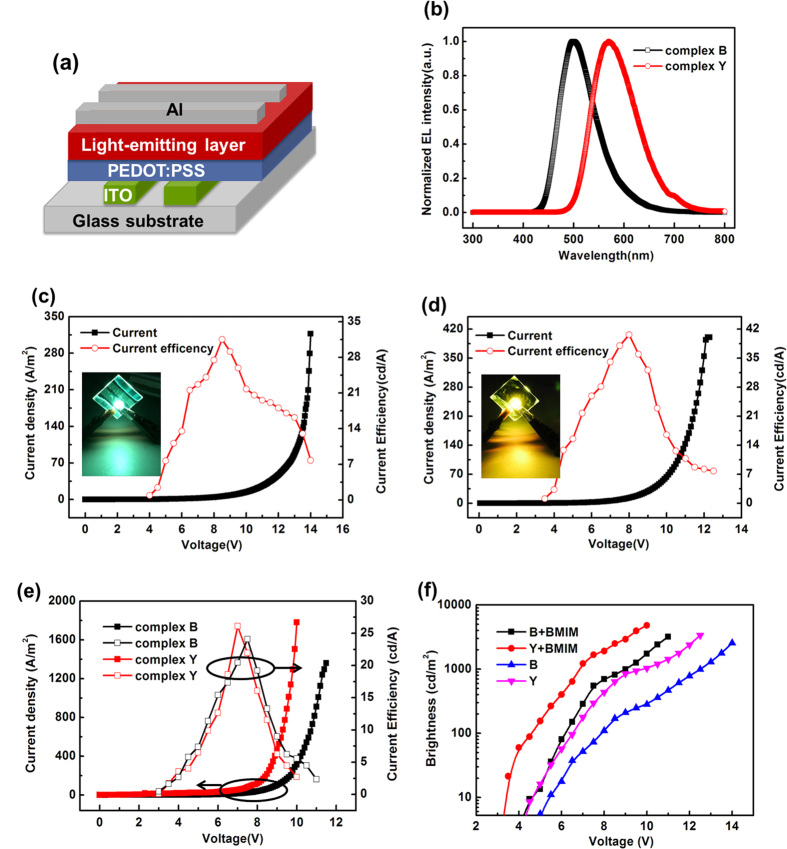
(**a**) As-fabricated device configuration. (**b**) Normalized EL spectra of LECs device. (**c**) Current intensity and current efficiency versus voltage characteristics of the blue-green-emitting LECs, the inset is the optical photo of the luminous LEC. (**d**) Current intensity and current efficiency versus voltage characteristics of the yellow-emitting LECs, the inset is the optical photo of the luminous LEC. (**e**) Current intensity and current efficiency versus voltage characteristics of the device with BMIM-PF_6_. (**f**) Luminance-voltage characteristics of the devices.

Figure 2b shows the EL spectra of the LECs based on complexes **B** and **Y**, with the EL peaks at 499 nm and 570 nm, respectively. These EL spectra are in agreement with those of the neat film PL spectra of the cationic Ir(III) complexes (Fig. 1b). Figure 2c,d show the device current intensity and current efficiency as a function of the applied voltage, and the inset is the optical photo of the luminous LECs with complex **B** and complex **Y** at 10 V, respectively. Turn on voltage (brightness of 1 cd m^−2^ in 1s) of the two LECs are 4.5 V and 3.5 V, which are lower than previously reported LECs based on Ir-iTMCs without BMIM-PF_6_^26^,^27^,^28^. Moreover, we achieved peak current efficiency, power efficiency and external quantum efficiency of 31.6 cd A^−1^, 11.7lm W^–1^ and 9.9% for the blue-green-emitting LECs device at 8.5 V and 40.6 cd A^−1^, and 16.1lm W^–1^, 12.2% for yellow-emitting LECs devices at 8 V, which are rarely reported so far. In order to reduce the turn on voltage and response time (the time required to reach the maximum brightness) of the LECs, an ionic liquid BMIM-PF_6_ was added into the light emitting layer and mass ratio of complex and BMIM-PF_6_ was 5:1. Figure 2e shows the current density-voltage and current efficiency-voltage characteristics of the blue-green and yellow LECs with BMIM-PF_6_. High current efficiencies of 24.1 cd A^−1^ for the blue-green-emitting LECs was obtained at 7.5 V and 27.3 cd A^−1^ for yellow-emitting LECs with BMIM-PF_6_ was obtained at 7 V. When the ionic liquid was added to LECs, the turn on voltage of device based on complex **B** decreased from 4.5 V to 3 V, and the current density significantly increased because of the improved carrier injection with the higher density of mobile ions. The brightness and current efficiency of the device increased from 19 cd/m^2^ to 80 cd/m^2^ and from 13.5 cd A^−1^ to 15.4 cd A^−1^, respectively, under the same bias voltage of 6 V. Despite the high carrier injection, due to a faster propagation of the doped zones leading to more extensive exciton quenching and the mismatch of carrier, the current efficiency of the device without BMIM-PF_6_ was higher at the whole. For LECs device based on complex **Y**, the turn on voltage of device decreased from 3.5 V to 3 V; at the same time, the brightness of the device increased from 61.8 cd/m^2^ to 361 cd/m^2^ at 6 V; but current efficiency decreased from 25.8 cd A^−1^ to 12.6 cd A^−1^ (6 V) due to carrier mismatch.

**Figure 3:**
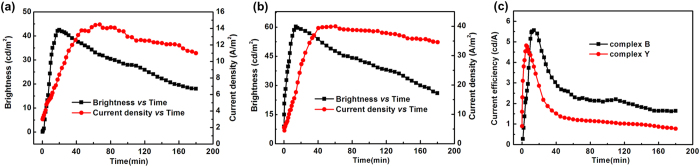
(**a**) Time-dependent curves of current density and brightness of complex **B** LECs with BMIM-PF_6_ at 3.5 V bias. (**b**) Time-dependent curves of current density and brightness of complex **Y** LECs with BMIM-PF_6_ at 3.5 V bias. (**c**) Time-dependent curves of current efficiency of complexes **B** and **Y** LECs with BMIM-PF_6_ at 3.5 V bias.

As shown in Fig. 3, at a constant voltage, the current density, brightness and current efficiency of LECs with BMIM-PF_6_ increase with time first, then they start to decay gradually after reaching its maximum value. With time increasing, the applied voltage causes more anions accumulate at the anode interface and deplete at the cathode. The carrier injection and recombination was facilitated, leading to the increased current density and brightness of LECs devices. While the current density was further rising, the brightness decay was attributed to unbalanced carrier injection. With current density dropping, the brightness decayed faster probably due to the water assisted decomposition of the cationic Ir(III) complex and the subsequent formation of luminescence quenchers^29^. The current efficiencies of 1.9 cd A^−1^ and 1.92 cd A^−1^ at 3.5 V for blue-green and yellow LECs were shown on Fig. 2e, which was consistent with the measured within 2 mins. With the facilitating of the carrier injection and recombination, the maximun current efficiency of 5.5 cd A^−1^ and 4.8 cd A^−1^ were achieved. The devices without BMIM-PF_6_ were also evaluated at 5 V, and detailed electrical characteristics of the LECs are summarized in Table 2. Stability is an essential factor of virtue in electroluminescent devices, while lifetimes of most of LECs range from a few hours to a few days. Now, most of the work on the stability problem has been focused on improving the design of the complexes. For example, the use of bulky substituents on Ir-iTMCs leads to more stable LECs, probably through enhanced hydrophobicity of the complexes that limits the occurence of water induced substitution reactions. The studies also demonstrated that Ir-iTMCs with the intramolecular π-π interactions can reduce the degradation reaction in metal-centered states to some extent, which were beneficial to improve the stability of the devices.

**Figure 4:**
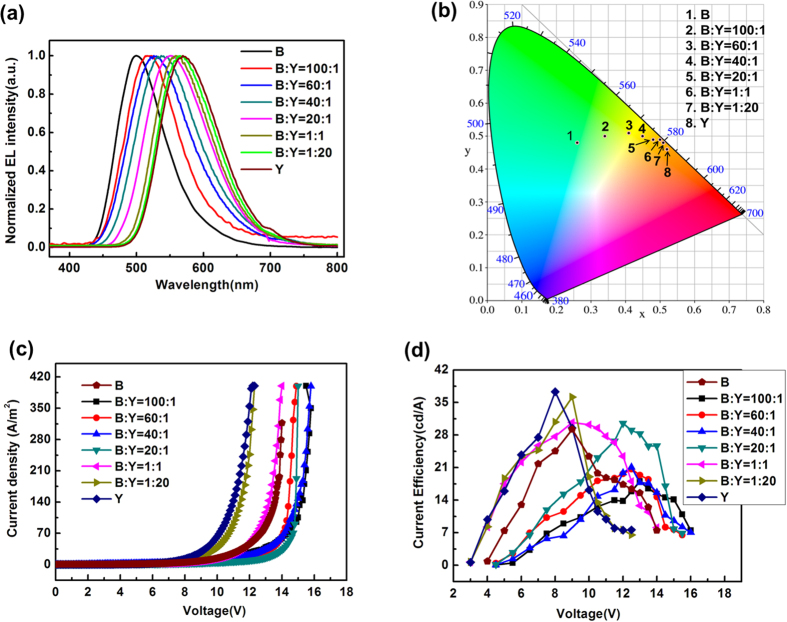
(**a**) EL spectra of the LECs devices with varied mixed ratio. Mass ratio of **Y** and **B** was varied to precisely control the color of emitting light. (**b**) CIE coordinates of the devices with varied mixed ratio. (**c**) The current density-voltage characteristics of the LECs. (**d**) The current efficiency-voltage characteristics of the LECs.

LECs with the structure of ITO/PEDOT:PSS/light emitting layer (**B**:**Y**)/Al were fabricated with various ratio of complex **B** to complex **Y**. Generally, the device with the light-emitting layer composed of two luminescent materials has a couple of emission peaks whose relative intensities can be adjusted according to the mixed proportion. However, EL spectra (shown in Fig. 4a) of the LECs and PL spectra (Fig. S3, Supporting Information) of flims in our study with varied mixed ratio of two cationic iridium complexes show only a single tunable emission peak. The relationship between the mass ratio of each complex and the peak of EL spectra appear to be directly proportional, clearly indicating that the relative emission from the two blending complexes can be readily controlled by varying their mass ratio. The possible mechanism for the tunable EL spectra may be due to the rigidochromic shift of complex **Y** and the energy transfer from complex **B** to complex **Y**. Firstly, a large overlap between the phosphorescence spectrum of complex **B** and the absorption band of complex **Y** suggest a possible efficient triplet energy transfer from complex **B** to complex **Y**, which has been confirmed by the dominant emission from complex **Y** shown in the EL spectra. On the other hand, photophysical measurements have suggested that the phosphorescence emission of complex **Y** originate from the dominant ^3^MLCT and ^3^LLCT state, and it thus suffer the rigidochromic effect from 298 to 77 K. A blue-shift can be observed for a charge transfer emission in a more rigid matrix due to a perturbed dipolar relaxation of the charge transfer transition state^30^,^31^. To better understand this phenomenon, PL measurements of complex **Y** in PVK film were carried out. It can be observed from Fig. S3 (Supporting Information) that the PL spectra for complex **Y** in PVK films show obvious blue-shift from 571 to 520 nm with reducing its concentration to 2 wt% in the film. The PVK (rigid matrix) films and photophysical results presented in the films lead us to suppose that the blue-shifts observed here are probably relate to the rigidochromic effect in complex **Y**. The magnitude of the rigidochromic shift is also related to the degree of MLCT character in the excited state^32^. With the increase of complex **Y** concentration, a gradual redshift from the blue-green emission to the yellow emission is observed. No intrinsic emission of two iridium complexes observed in the EL spectrum of devices suggests that the complete energy transfer from complex **B** to complex **Y** and the rigidochromic shift of complex **Y** have been realized simultaneously.

**Figure 5:**
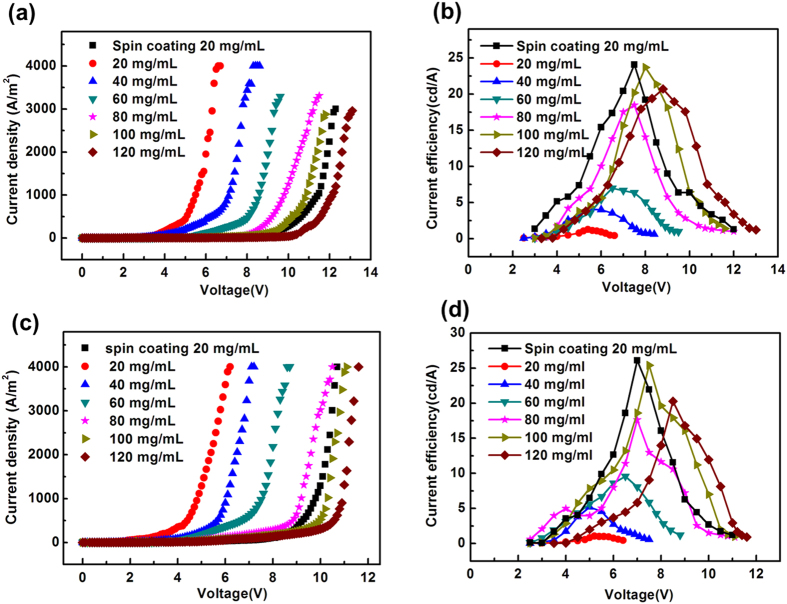
(**a**) The characteristics of current density versus applied voltage of LECs device with complex **B**. (**b**) The characteristics of current efficiency versus applied voltage of devices with complex **B**. (**c**) The characteristics of current density versus applied voltage of LECs device with complex **Y**. (**d**) The characteristics of current efficiency versus applied voltage of devices with complex **Y**.

Adding a small amount of complex **Y** into complex **B** (**B**:**Y** = 100:1), EL peak of the device exhibited at 516 nm (shown in Table 3), which is significantly different from that of pure complex **B** at 499 nm. Turn on voltage of 5 V is close to that of pure complex **B** device, but the peak current efficiency is much smaller than that of complex **B**. The current efficiency of the devices is mainly determined by the photoluminescence quantum yields (PLQY) of the iTMC emitters^33^. The luminescence of the doped system mainly comes from complex **Y**, and the low concentration of complex **Y** leads to the decrease of PLQY in the films. With the increasing of complex **Y** concentration, turn on voltage decreased and the efficiency is gradually increasing (shown in Fig. 5c,d). With the large amount of complex **Y** (**B**:**Y** = 1:20), EL spectra peak of the device exhibited at 567 nm close to the spectra of pure complex **Y**. Detailed optoelectronic characteristics of the LECs are summarized in Table 3. With the increase of the mass ratio of complex **Y**, the turn on voltage of the devices drops, while the current density, maximum current efficiency increase. The resulting LECs still remain high efficiencies in the range from 19.7 cd A^−1^ to 39.1 cd A^−1^.

LECs (5 × 5 mm^2^) with the complex solution concentration of 20, 40, 60, 80, 100, 120 mg mL^−1^ in CH_3_CN were fabricated with doctor-blade coating method. Figure 5 shows the current density and current efficiency curves of the devices with varying the solution concentration of cationic iridium complex **B** and complex **Y**. With 20 mg mL^−1^ by blade coating, the current density increased sharply with voltage and the breakdown voltage was 6.2 V and 5.2 V, while that of spin coating was 11.8 V and 10.2 V for complex **B** and complex **Y**, respectively. However, the peak current efficiencies obtained by blade coating were 1.2 cd A^−1^ and 1.0 cd A^−1^, which were considerably smaller than the devices prepared by spin coating with 24.0 cd A^−1^, 26.1 cd A^−1^ for complex **B** and complex **Y**, respectively. The thickness of the emission layer with blade coating was very different from that with spin coating at the same concentration, because the film thickness was influenced by many factors, such as surface tension of solution, blading speed, substrate surface energy, and substrate temperature and so on. The current efficiency, maximum luminance and breakdown voltage increased with the complex concentration. When the complex concentration reached 100 mg/ml, the peak current efficiencies of 23.7 cd A^−1^, 25.4 cd A^−1^ were comparable to that obtained by spin coating of 20 mg mL^−1^, while dropped to 20.3 cd A^−1^, 20.7 cd A^−1^ with increasing concentration to 120 mg mL^−1^ for complex **B** and complex **Y**, respectively.

**Figure 6:**
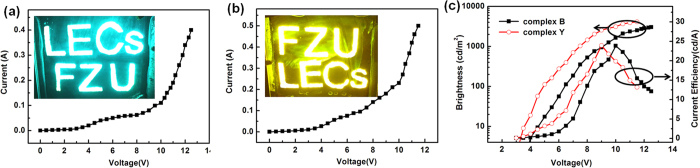
(**a**) Current versus characteristics of 4 inches blue-green-emitting LECs device, the inset is the optical photo of the 4 inches luminous LECs (at 10 V). (**b**) Current versus characteristics of 4 inches yellow-emitting LECs device, the inset is the optical photo of the 4 inches luminous LECs (at 10 V). (**c**) Average luminance-voltage and current efficiency-voltage characteristics of the devices.

To demonstrate the extensibility of our blade coating method, LECs with large size (8 × 6 cm^2^, 4 inches) doctor-blade coated films were fabricated. The concentration of cationic iridium complex **B** and complex **Y** was kept at 100 mg mL^−1^ in CH_3_CN solution. Uniform light blue-green and yellow emissions were obtained with maximum average luminance of 3027 cd m^−2^ and 4120 cd m^−2^ and peak current efficiency of 23.8 cd A^−1^ and 24.5 cd A^−1^ (Fig. 6c), respectively. Here, average luminance was defined as the average value of the measured brightness of six different positions on the 4 inches light emitting device. These results proved the feasibility of doctor-blade coating in the fabrication of large area devices, which laid the foundation for the practical application of LECs.

## Conclusions

In conclusion, highly efficient blue-green and yellow light LECs with simple sandwich structure based on Ir(III complexes **B** and **Y** were fabricated by using solution processes. Peak current efficiency as high as 31.6 and 40.6 cd A^−1^ were obtained for the blue-green and yellow LECs, respectively. By blending complex **B** with complex **Y** in the light emitting layer, tunable emissions with a single spectrum peak were obtained which can be easily controlled by varying the two complexes mass ratio. Efficient LECs were also fabricated with simple doctor-blade coating. The results confirm that the active layer thickness is an important factor for the enhancement of LECs performance. For the first time, we reported a 4 inches uniform light emission from LECs by doctor-blade coating. These results proved the feasibility of doctor-blade coating in the fabrication of large area devices, which laid the foundation for the practical application of LECs.

## Additional Information

**How to cite this article**: Zeng, Q. *et al*. Large Size Color-tunable Electroluminescence from Cationic Iridium Complexes-based Light-emitting Electrochemical Cells. *Sci. Rep.* **6**, 27613; doi: 10.1038/srep27613 (2016).

## Supplementary Material

Supplementary Information

## Figures and Tables

**Table 1:** Photophysical and electrochemical characteristics of complexes B and Y.

	Room-temperature emission			Emission at 77 K	Electrochemical data^c^	
	Solution *λ*[nm]^a^	Neat film *λ*[nm]	*Φ*_em_ (*τ*[μs])^b^	*λ*[nm]	*E*_*ox*_^1/2^(V)	*E*_*red*_^1/2^(V)
B	516	499	0.37 (0.8)	478	1.16	−1.92
Y	586	571	0.48 (0.5)	518	0.79	−1.96

^a^In CH_3_CN solutions.

^b^Measured in the neat film.

^c^The data were versus Fc^+^/Fc.

**Table 2:** Electrical characteristics of the LECs based complexes **B** and **Y**.

Complex	Bias [V]	t_on_^a^ [s]	t_max_^b^ [min]	t_1/2_^c^ [min]	B_max_[cd m^−2^]	EL λ[nm]	CIE (x, y)
B	5	30	42	165	55	499	(0.27, 0.46)
Y	5	5	25	175	215	569	(0.51, 0.47)
B+BMIM	3.5	15	20	145	42.5	500	(0.27, 0.47)
Y+BMIM	3.5	2	13	160	60.3	570	(0.51, 0.48)

The device structure consists of ITO/PEDOT:PSS/complex or complex: BMIM-PF_6_(5:1)/Al.

^a^Time required to reach 1 cd/m^2^.

^b^Time required to reach the maximum brightness (response time).

^c^Time to reach half of the maximum luminance.

**Table 3:** Optoelectronic characteristics of color-tunable LECs.

Ratio of B and Y	Turn on voltage (V)	EL (nm)	Maximum efficiency (cd A^−1^) (@voltage)	CIE (x, y)
D1: B	4.5	499	31.5 (8.5 V)	(0.26, 0.48)
D2: B:Y = 100:1	5	516	19.7 (13 V)	(0.34, 0.49)
D3: B:Y = 60:1	5	525	22.4 (12.5 V)	(0.41, 0.51)
D4: B:Y = 40:1	5	537	22.9 (12.5 V)	(0.45, 0.50)
D5: B:Y = 20:1	4.5	546	33.1 (12 V)	(0.48, 0.49)
D6: B:Y = 1:1	3.5	558	33.5 (9 V)	(0.50, 0.49)
D7: B:Y = 1:20	3.5	567	39.1 (9 V)	(0.51, 0.48)
D8: Y	3.5	570	40.6 (8 V)	(0.52, 0.48)

The device structure consists of ITO/PEDOT:PSS/**B**:**Y**/Al, where **B**:**Y** denotes the molar ratio between complex **B** and complex **Y**. Turn on voltage required to reach 1 cd/m^2^. Maximum efficiency of varying ratio devices showed at different voltages.
