# Supplementary material for: Large Size Color-tunable Electroluminescence from Cationic Iridium Complexes-based Light-emitting Electrochemical Cells
Source: Sci Rep. 2016 Jun 9;6:27613. doi: 10.1038/srep27613 (PMC4899800; doi:10.1038/srep27613)
Supplement: Supplementary Information [file srep27613-s1.pdf]

Large Size Color-tunable Electroluminescence from Cationic Iridium Complexes-based Light-emitting Electrochemical Cells

Qunying Zeng[1], Fushan Li[1,*], Tailiang Guo[1], Guogang Shan[2,*], Zhongmin Su[2]

[1]Institute of Optoelectronic Technology, FuzhouUniversity, Fuzhou 350002, People's Republic of China

[2]Institute of Functional Material Chemistry, Faculty of Chemistry, Northeast Normal University, Changchun, Jilin 130024, People's Republic of China

Supporting Information

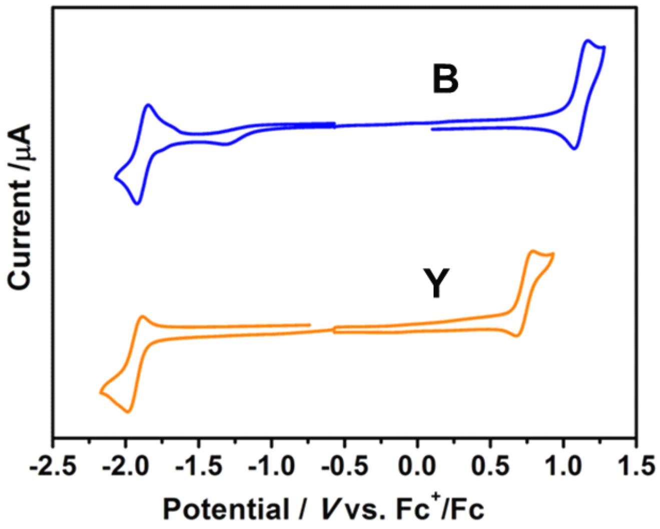

Figure S1 cyclic voltammogram of complexes B and Y.

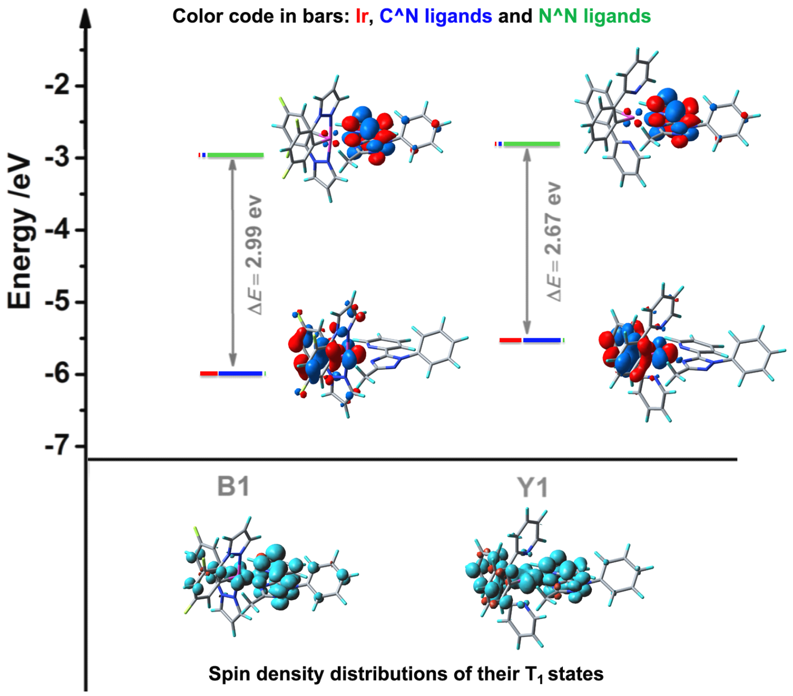

Figure S2 Calculated HOMO and LUMO orbitals for complexes B and Y.

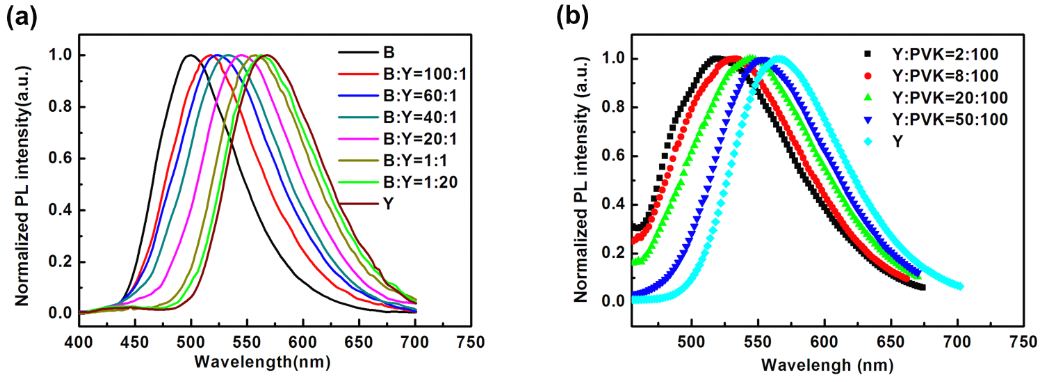

Figure S3 (a) PL spectra of the films with varied mixed ratio. (b) PL spectra of complex Y doped in PVK films.
